# Subperiosteal/subgaleal drainage vs. subdural drainage for chronic subdural hematoma: A meta-analysis of postoperative outcomes

**DOI:** 10.1371/journal.pone.0288872

**Published:** 2023-08-01

**Authors:** Chunhui Chen, Yu Xiong, Xinyue Huang, Xiumei Guo, Xiaodong Kang, Jianfeng Zhou, Zhigang Pan, Hanlin Zheng, Shuni Zheng, Linxing Wang, Weipeng Hu, Liming Zhuang, Feng Zheng

**Affiliations:** 1 Department of Neurosurgery, the Second Affiliated Hospital, Fujian Medical University, Quanzhou, Fujian Province, China; 2 Department of Neurology, the Second Affiliated Hospital, Fujian Medical University, Quanzhou, Fujian Province, China; 3 Division of Public Management, the Second Affiliated Hospital, Fujian Medical University, Quanzhou, Fujian Province, China; 4 Department of Pharmacy, the Second Affiliated Hospital, Fujian Medical University, Quanzhou, Fujian Province, China; Duke University Medical Center: Duke University Hospital, UNITED STATES

## Abstract

**Background:**

Chronic subdural hematoma (CSDH) is commonly treated via surgical removal of the hematoma, placement of a routine indwelling drainage tube, and continuous drainage to ensure that the blood does not re-aggregate following removal. However, the optimal location for placement of the drainage tube remains to be determined.

**Objectives:**

To aid in establishing a reference for selecting the optimal method, we compared the effects of different drainage tube placements on CSDH prognosis via a systematic review and meta-analysis of previous clinical studies.

**Data sources:**

PubMed, Embase, and Cochrane databases.

**Study eligibility criteria:**

We searched for clinical studies comparing the outcomes of subperiosteal/subgaleal drainage (SPGD) and subdural drainage (SDD) for CSDH published in English prior to April 1, 2022.

**Participants:**

The final analysis included 15 studies involving 4,318 patients.

**Results:**

Our analysis of the pooled results revealed no significant differences in recurrence rate between the SDD and SPGD groups. We also observed no significant differences in mortality or rates of postoperative complications (infection, pneumocephalus, or epilepsy) between the SDD and SPGD groups.

**Conclusions:**

These results suggest that the choice of SDD vs. SPGD has no significant effect on CSDH prognosis, highlighting SPGD as an alternative treatment option for CSDH.

## Introduction

Chronic subdural hematoma (CSDH), a type of intracranial hematoma that occurs mainly in older adults [1, 2], is pathogenically characterized by the slow accumulation of blood between the cerebral cortex and dura mater [3, 4]. Clinical manifestations of CSDH vary depending on the size of the hematoma and the extent to which it compresses different parts of the brain, although common features include headache, limb weakness, mental disorders, and epilepsy [5, 6]. Previous studies have reported a CSDH incidence as high as 20.6/100,000 cases per year, with vascular injury due to head trauma representing the main cause of bleeding in 75% of cases [7, 8].

At present, the most common treatment strategy for CSDH involves surgical removal of the hematoma, placement of a routine indwelling drainage tube, and continuous drainage to ensure that the blood does not re-aggregate in the space left following removal of the hematoma [9, 10]. Research has demonstrated that the use of a conventional drainage tube can significantly reduce the rate of CSDH recurrence when compared with treatments implemented without a drainage tube [11, 12]. Despite the recent utilization of subperiosteal or subgaleal drainage (SPGD), subdural drainage (SDD) remains the most common strategy [13], and the optimal drainage location remains to be determined [14, 15]. To aid in establishing a reference for selecting the optimal drainage method, the present study aimed to compare the effects of different drainage tube placements on prognosis in patients with CSDH via a systematic review and meta-analysis of previous clinical studies.

## Materials and methods

### Search strategy

We searched the PubMed, Embase, and Cochrane databases for articles reporting the results of clinical studies comparing the outcomes of SPGD and SDD for CSDH (A complete search strategy is attached to the online material). Initially, all relevant studies published in English prior to April 1, 2022 were included based on a search conducted using the appropriate keywords (“drain”, “drainage”, “chronic subdural hematoma”) and MeSH terms. The search was restricted to RCTs and observational cohort studies. The reference lists of the included studies were further reviewed to ensure inclusion of other relevant clinical studies.

### Selection criteria

Articles were independently retrieved and screened by two researchers (C. C. and Y. X.), and any differences in eligibility assessments were resolved through discussion. After excluding articles unrelated to the study topic based on titles and abstracts, two researchers (C. C. and Y. X.) read the full text of the remaining articles, which were then screened based on the following inclusion criteria: (a) inclusion of patients diagnosed with CSDH, (b) treatment strategy consisting of surgery and postoperative drainage, (c) clear distinction between SDD and SPGD for postoperative drainage, (d) availability of the full text and sufficient data. Exclusion criteria were as follows: (a) population consisting of patients without CSDH, (b) treatment involving non-surgical removal of the hematoma or surgical removal without catheter drainage, (c) incomplete or unextractable data, (d) no clear distinction between SDD and SPGD for postoperative drainage, (e) non-relevant study type (e.g., case reports, letters, comments, summaries of meetings, editorials, programs, guidelines, and animal research articles).

### Data extraction and quality assessment

Two independent investigators (C. C. and Y. X.) extracted the data and assessed the quality of the studies using standardized tables that included year, author, trial design, inclusion criteria, and outcome measures. Missing data were obtained from the original author whenever possible. All differences were resolved through team discussion and evaluation. The Cochrane Collaboration’s Risk of Bias tool [16] and the Newcastle–Ottawa Scale [17] were used to assess the quality of RCTs and cohort studies, respectively. The Cochrane Risk of Bias tool evaluates the quality of RCTs across seven domains (allocation concealment, random sequence generation, blinding of participants and personnel, blinding of outcome assessment, data integrity, selective reporting, and other sources of bias). The maximum total score given by the Newcastle–Ottawa Scale is 9 points across the three categories of selection, comparability, and outcome (maximum scores of 4, 2, and 3 points, respectively). Scores of 7 or higher, 4–6, and 0–3 points correspond to “high quality”, “moderate”, and “weak”, respectively. The scores of the six observational cohort studies ranged from 7 to 8, indicative of generally high methodological quality.

### Outcome measures

The primary outcome measures were the postoperative recurrence rate and mortality. Secondary outcome measures included postoperative infection, postoperative epilepsy and intracranial hematoma. Because pneumocephalus may be associated with adverse outcomes [18] it was also included as a secondary outcome in the present study, defined as the presence of intracranial gas on radiological findings.

### Statistical analysis

RevMan5.3 was used to perform this meta-analysis of studies comparing the effects of SPGD and SDD on recurrence rate, mortality, postoperative infection, postoperative epilepsy, pneumocephalus, and intracranial hematoma in patients with CSDH. Outcomes were compared in terms of odds ratios (ORs), 95% confidence intervals, and P values for dichotomous variables. In addition, a random-effects model was used to pool the results of the primary studies. Heterogeneity was assessed using the I^2^ test, and an I^2^ ≤ 50% was considered to indicate acceptable heterogeneity as specified in the Cochrane manual.

## Results

**Fig 1:**
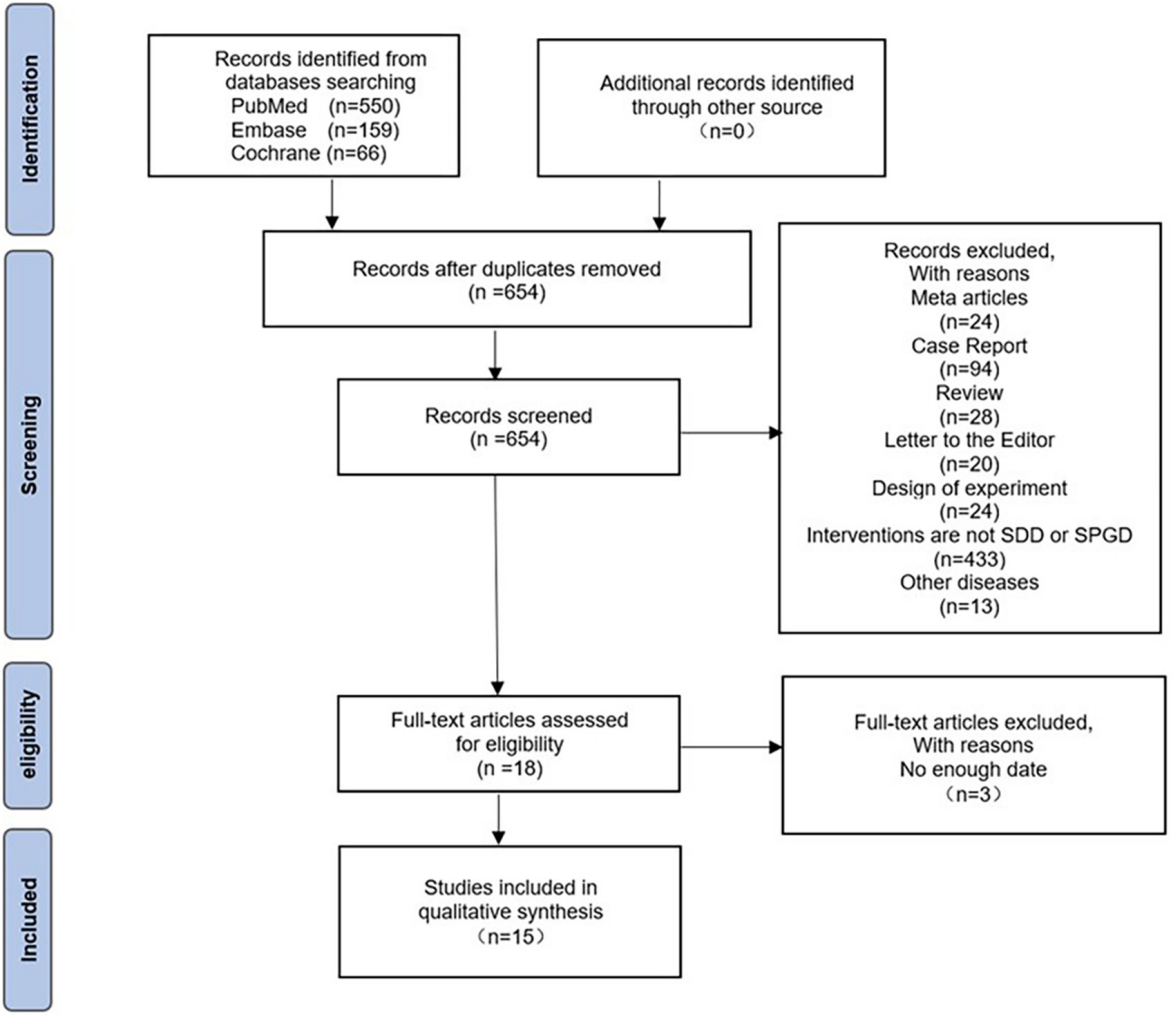
Flow chart depicting the search strategy and selection process. **SDD:** Subdural drainage; **SPGD:** Subperiosteal or subgaleal drainage.

**Fig 2:**
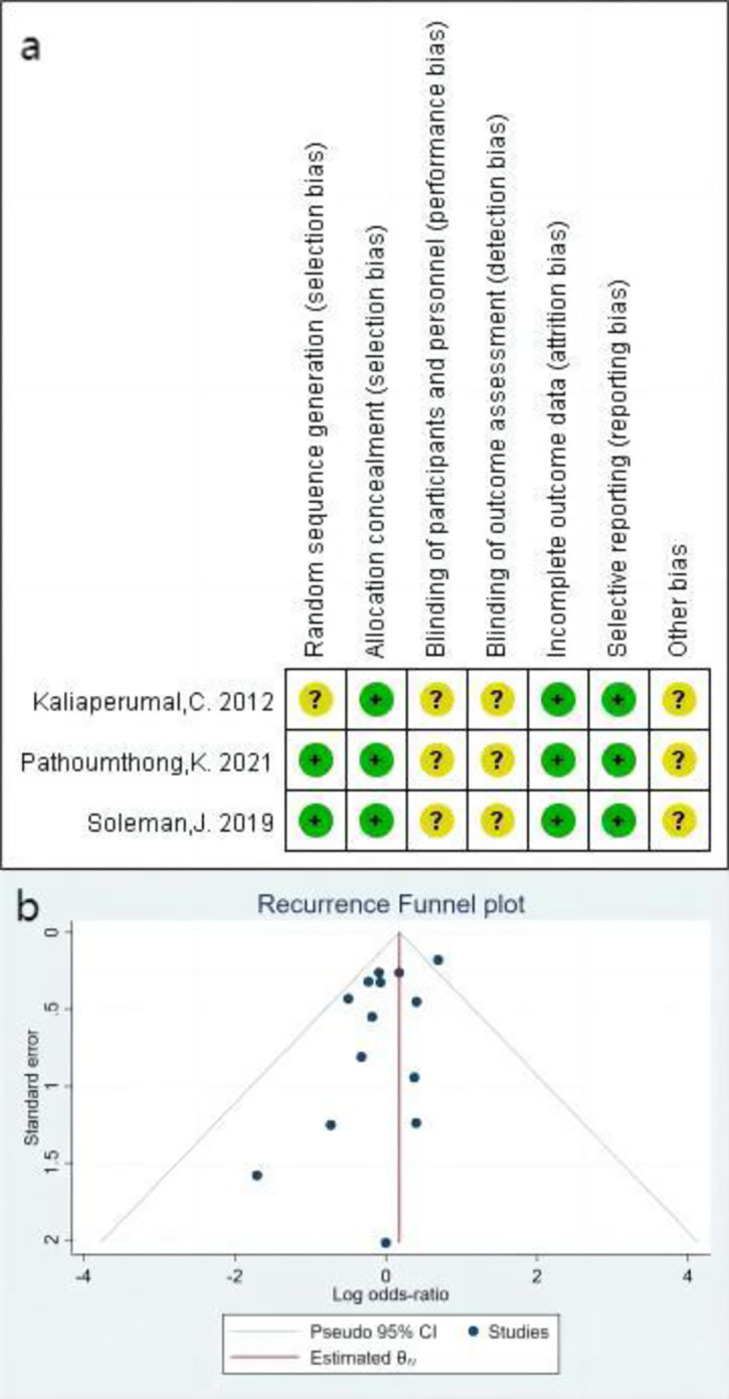
(a). Risk of bias for randomized controlled trials in the present analysis. (b) Funnel plot of publication bias for recurrence rates. M-H: Mantel–Haenszel statistic. CI: Confidence interval.

In total, 775 studies were retrieved based on the initial search strategy. After removing duplicates, 654 articles remained. Among them, 636 were excluded based on a review of the titles and abstracts. After further review, an additional three articles were excluded due to insufficient data. Thus, 15 studies [9, 10, 14, 15, 19–29] encompassing 4,318 patients were included in the final analysis. There were 2,547 patients in the SDD group and 1,985 patients in the SPGD group. The average follow-up time was 6.58 months. Three of these studies were randomized controlled trials (RCTs), while the remaining 12 were cohort studies. The detailed search strategy and selection process are shown in Fig 1. The characteristics of the included studies are presented in Table 1 (the complete NOS chart has been uploaded as online material) and Fig 2A.

**Table 1:** Baseline characteristics of the fifteen included studies.

Study	Year	Study type	Patients	Group		Mean age		Gender (Male/Female)		Drain-duration(h)	Inclusion time	Follow-up times(m)	NOS	Surgical technique	MMA embolization	Steroid drugs	Selection of drainage methods
				SDD	SPGD	SDD	SPGD	SDD	SPGD								
Hwang et al. [22]	2022	RCS	262	203	59	69.8	71.4	137/66	47/12	24-48h	2015.01–2019.12	12	7	BH	NA	NA	SD
Singh et al. [19]	2021	PCS	70	35	35	60.2	59.4	22/13	22/13	32.91(SDD)、34.6(SPGD)	2016.02–2017.07	6	6	BH	NA	NA	RD
Pathoumthong et al. [20]	2021	RCT	42	21	21	NA	NA	12/9	16/5	48	2019.04–2020.05	6	NA	BH	NA	No	RD
Kamenova et al. [21]	2020	RCS	361	290	171	77.7	77.3	208/82	114/57	48	2013.01–2017.11	2	4	BH	NA	NA	NA
Gazzeri et al. [23]	2020	RCS	414	382	146	NA	NA	205/107	69/33	48–72	2011.09–2017.09	NA	7	BH/C	NA	NA	SD
Zhang et al. [14]	2019	RCS	570	329	241	71.0	70	251/78	171/70	24–96	2010–2017	24	7	BH	NA	NA	SD
Soleman et al. [9]	2019	RCT	220	100	120	81.0	78.0	68/32	81/39	48	2013.04–2015.12	12	NA	BH	NA	NA	RD
Häni et al. [27]	2019	RCS	349	135	214	74.10	72.94	84/51	150/64	24–48	2012.06–2016.08	6	6	BH	NA	NA	SD
Lancz et al. [28]	2019	PCS	577	533	44	NA	NA	NA	NA	24–48	2013.05–2014.01	2	5	BH	NA	NA	NA
Sjåvik et al. [24]	2017	RCS	1094	330	764	74.0	74	249/81	518/246	12-18(SDD)、24(SPGD)	2005.01–2010.12	6	8	BH	NA	NA	SD
Ishfaq et al. [26]	2017	PCS	62	31	31	73.0	72	19/12	22/9	72–96	2015.07–2016.06	NA	6	BH	NA	NA	SD
Chih et al. [15]	2017	PCS	60	30	30	70.0	68	20/10	21/9	24	2012.01–2014.01	3	5	BH	NA	NA	SD
Oral et al. [10]	2015	RCS	74	38	36	66.1	68.1	28/8	29/9	48–72	2009.01–2011.12	3	6	BH	NA	NA	RD
Kaliaperumal et al. [25]	2012	RCT	50	25	25	NA	NA	16/9	17/8	48	NA	6	NA	BH	NA	NA	SD
Bellut et al. [29]	2012	RCS	113	65	48	71.0	71	45/20	32/16	48	2007–2009	3	7	BH	NA	NA	SD

NOS, Newcastle–Ottawa Quality Assessment Scale; NA, not applicable; SPGD, subperiosteal or subgaleal drainage; SDD, subdural drain; RCT, randomized controlled trial; RCS, retrospective cohort study; PCS, prospective cohort study; h, hours; m, months; y, years; C, craniotomy; BH, burr hole; MMA: middle meningeal artery; SD, Surgeon decision; RD, Random.

### Recurrence

**Fig 3:**
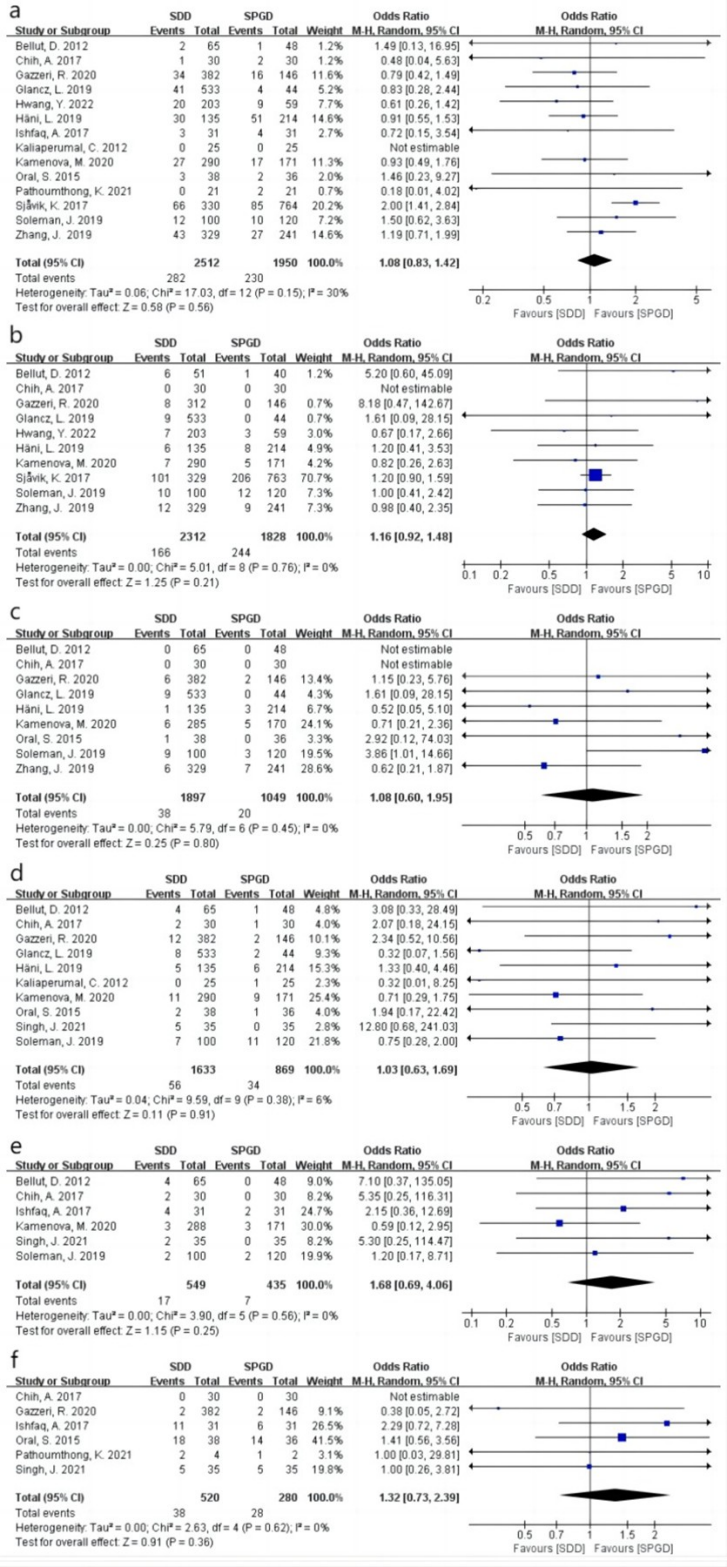
Forest plots showing complications for the SDD and SPGD groups. (a): Recurrence. (b) Mortality rate. (c) Postoperative infection. (d) Postoperative epilepsy. (e) Intracranial hematoma. (f) Pneumocephalus. SDD: Subdural drainage. SPGD: Subperiosteal or subgaleal drainage. M-H: Mantel–Haenszel statistic. CI: Confidence interval.

**Fig 4:**
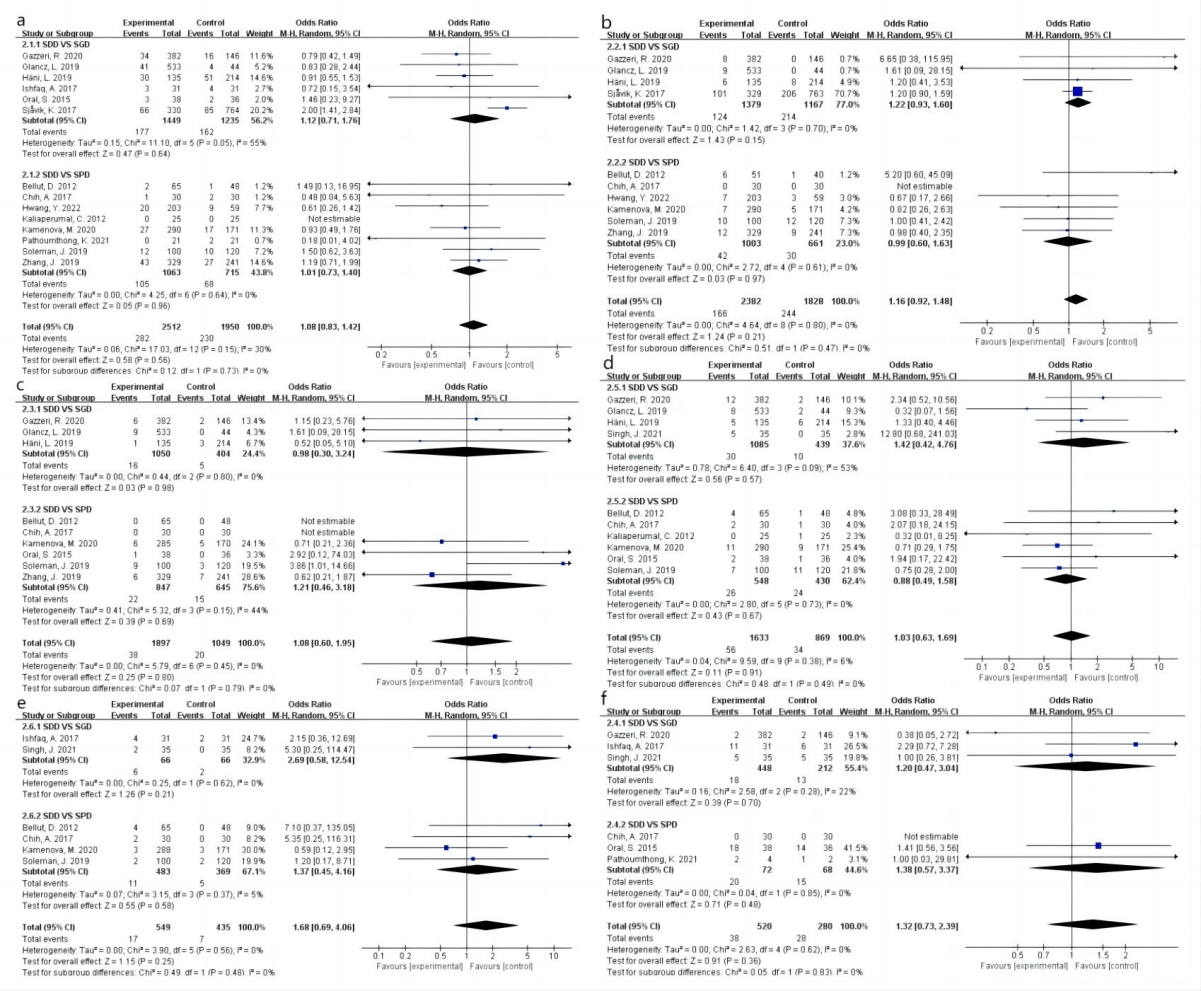
Forest plots showing the results of the subgroup analysis. (a): Recurrence. (b) Mortality rate. (c) Postoperative infection. (d) Postoperative epilepsy. (e) Intracranial hematoma. (f) Pneumocephalus. SDD: Subdural drainage. SPGD: Subperiosteal or subgaleal drainage. SPD: Subperiosteal drainage. SGD: Subgaleal drainage. M-H: Mantel–Haenszel statistic. CI: Confidence interval.

Across the 15 included studies [9, 10, 14, 15, 19–29], recurrence was reported in 512 patients at the end of follow-up. The analysis revealed no significant difference in the recurrence rate between the SDD and SPGD groups (odds ratio [OR]: 1.08 [95% confidence interval: 0.83, 1.42], I^2^ = 30%, P = 0.56) (Fig 3A). As more than 10 studies were included in the current analysis, publication bias was further analyzed using a funnel plot, which revealed no significant bias (Fig 2B). The SPDG group was then divided into subperiosteal drainage (SPD) and subgaleal drainage (SGD) subgroups to examine the effect of drainage tube placement on recurrence rate. No significant differences in recurrence were observed between the SPD (OR: 1.01 [0.73, 1.40], I^2^ = 0%, P = 0.96) and SGD groups (OR: 1.12 [0.71, 1.76], I^2^ = 55%, P = 0.64) (Fig 4A).

### Mortality rate

Across ten studies providing relevant data [9, 14, 15, 21–24, 27–29], 410 patients were reported to have died. There was no significant difference in mortality between the SDD and SPGD groups (OR: 1.16 [0.92, 1.48], I^2^ = 0%, P = 0.21) (Fig 3B). Further subgroup analysis revealed no significant difference in mortality between the SPD (OR: 0.99 [0.60, 1.63], I^2^ = 0%, P = 0.97) and SGD groups (OR: 1.22 [0.93, 1.60], I^2^ = 0%, P = 0.15) (Fig 4B).

### Postoperative infection

At the end of the follow-up period, nine studies reported postoperative infection in 58 patients [9, 10, 14, 15, 21, 23, 27–29]. An analysis of the pooled results revealed no significant difference in the rate of postoperative infection between the SDD and SPGD groups (OR: 1.08 [0.60, 1.95], I^2^ = 0%, P = 0.80) (Fig 3C). Subgroup analysis revealed no significant difference in infection rates between the SPD (OR: 1.21 [0.46, 3.18], I^2^ = 44%, P = 0.69) and SGD groups (OR: 0.98 [0.30, 3.24], I^2^ = 0%, P = 0.98) (Fig 4C).

### Postoperative epilepsy

Across 10 studies providing relevant data [9, 10, 15, 19, 21, 23, 25, 27–29], 90 patients were reported to have developed postoperative epilepsy, although there were no significant differences in the rate of postoperative epilepsy between the SDD and SPGD groups (OR: 1.03 [0.63, 1.69]; I^2^ = 6%; P = 0.91) (Fig 3D). Subgroup analysis also revealed no significant difference in the postoperative epilepsy rate between the SPD (OR: 0.88 [0.49, 1.58], I^2^ = 0%, P = 0.67) and SGD groups (OR: 1.42 [0.42, 4.76], I^2^ = 53%, P = 0.57) (Fig 4D).

### Intracerebral hematoma

The outcome of postoperative intracerebral hematoma was utilized to assess the resultant injury to brain parenchyma caused by the maneuvering of the drainage tube. Across six studies providing relevant data, intracerebral hematoma was reported in 24 patients [9, 15, 19, 21, 26, 29], and there was no significant difference between the SDD and SPGD groups (OR: 1.68 [0.69, 4.06], I^2^ = 0%, P = 0.25) (Fig 3E). Subgroup analysis also revealed no significant difference in the postoperative epilepsy rate between the SPD (OR: 1.37 [0.45, 4.16], I^2^ = 5%, P = 0.58) and SGD groups (OR: 2.69 [0.58, 12.54], I^2^ = 0%, P = 0.21) (Fig 4E).

### Pneumocephalus

The incidence of postoperative pneumocephalus was recorded in five studies [10, 15, 19, 20, 23], and an analysis of the pooled results revealed no significant difference in the incidence of pneumocephalus between the SDD and SPGD groups (OR: 1.32 [0.73, 2.39], I^2^ = 0%, P = 0.36) (Fig 3F). Subgroup analysis also revealed no significant difference in the postoperative epilepsy rate between the SPD (OR: 1.38 [0.57, 3.37], I^2^ = 0%, P = 0.48) and SGD groups (OR: 1.20 [0.47, 3.04], I^2^ = 22%, P = 0.70) (Fig 4F).

### Subgroup analysis based on RCTs

**Fig 5:**
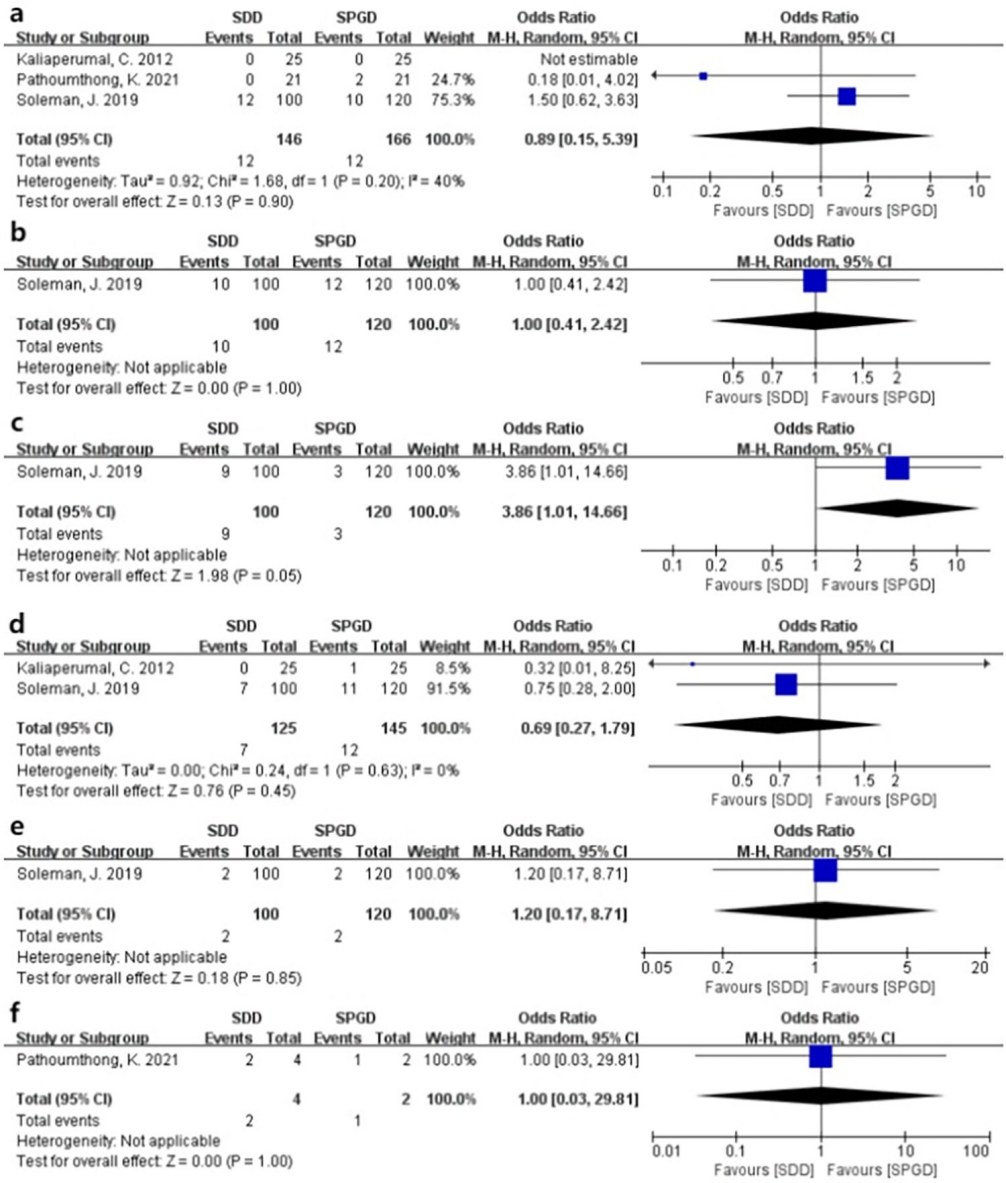
Subgroup analysis based on RCTs. (a) Recurrence. (b) Mortality rate. (c) Postoperative infection. (d) Postoperative epilepsy. (e) Intracranial hematoma. (f) Pneumocephalus. SDD: Subdural drainage. SPGD: Subperiosteal or subgaleal drainage. SPD: Subperiosteal drainage. SGD: Subgaleal drainage. M-H: Mantel–Haenszel statistic. CI: Confidence interval. RCT: Randomized controlled trial.

Subgroup analysis based on RCTs (Soleman et al., Pathoumthong et al., and Kariaperumal et al.) [9, 20, 25] showed that SPGD was associated with similar recurrence (OR: 0.89 [0.15, 5.39], I^2^ = 40%, P = 0.90), mortality (OR: 1.00 [0.41, 2.42], P = 1.00), seizure (OR: 0.69 [0.27, 1.79], I^2^ = 0%, P = 0.45), intracerebral hematoma (OR: 1.20 [0.17, 8.71], P = 0.85), pneumocephalus (OR: 1.00 [0.03, 29.81], P = 1.00), and postoperative infection (OR: 3.86 [1.01, 14.66], P = 0.05) compared to SDD (Fig 5).

## Discussion

**Fig 6:**
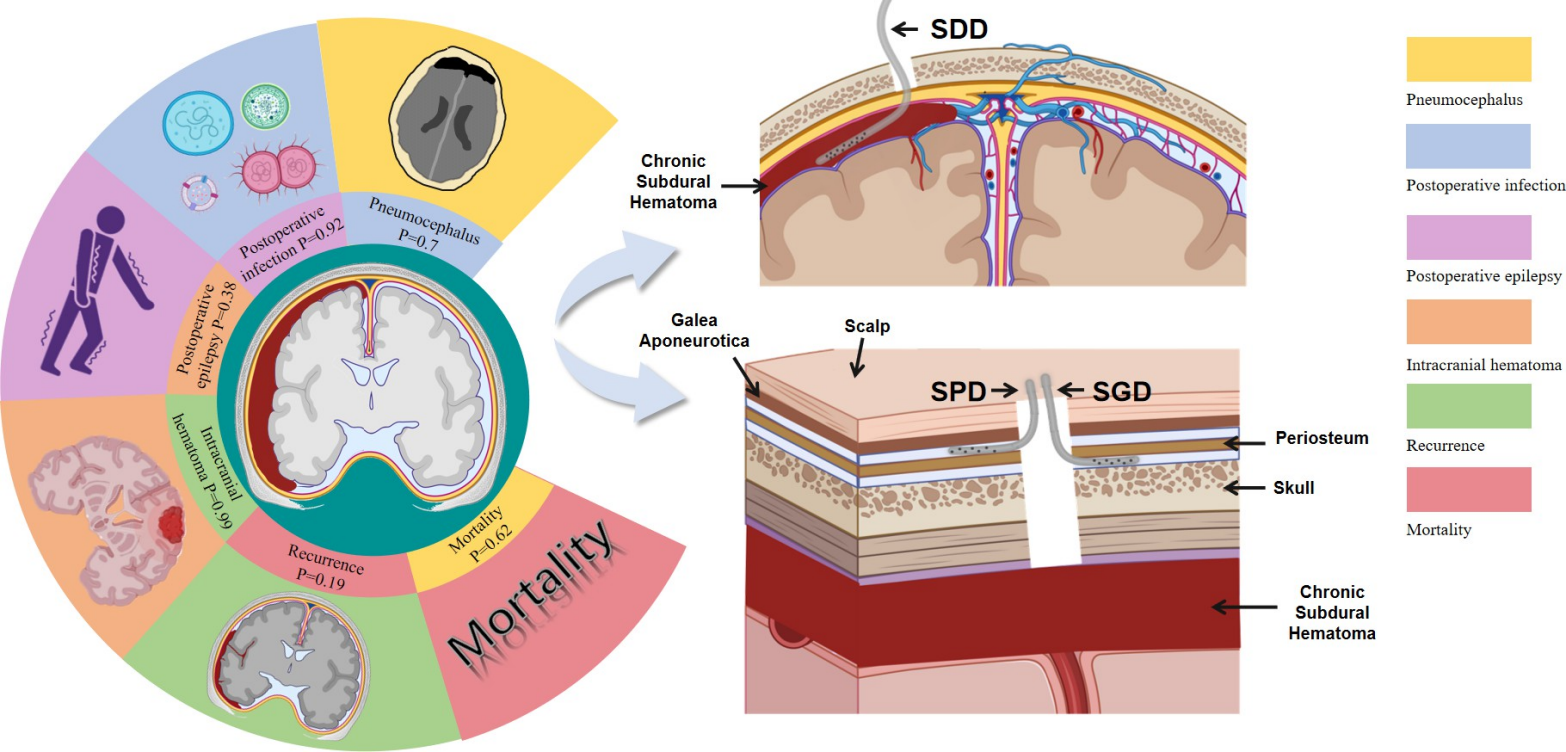
SDD and SPGD for chronic subdural hematoma. **SDD:** Subdural drainage; **SPGD:** Subperiosteal or subgaleal drainage.

In the current study, we analyzed the influence of different catheterization methods on the postoperative prognosis of patients with CSDH via a meta-analysis of 15 studies involving 4,318 patients. The advantages and disadvantages of different postoperative catheterization and drainage methods for CSDH were evaluated by integrating and analyzing several outcome indicators, including rates of recurrence, mortality, and postoperative complications (Fig 6). Given its significant impact on the prognosis of CSDH, the postoperative recurrence rate was selected as the primary outcome [30–33]. Our analysis of the pooled results revealed no significant differences in recurrence rate between the SDD and SPGD groups, suggesting that the choice of postoperative drainage method does not significantly impact the rate of CSDH recurrence. Our pooled analysis also revealed no significant differences in mortality or rates of postoperative complications (infection, pneumocephalus, or epilepsy) between the SDD and SPGD groups. These results further suggest that the drainage method does not influence postoperative outcomes.

CSDH is one of the most common neurosurgical conditions [13, 33]. While surgical removal of the hematoma is standard, the precise method used for postoperative drainage remains controversial [22, 34]. Several meta-analyses have reported a significantly lower recurrence rate of CSDH in patients treated with SPGD (SPD/SGD) than in patients treated with SDD [35–38]. However, in the present analysis, we observed no significant difference in recurrence rates between the SPGD and SDD group. This may be because only two of the 15 included studies [9, 24] reported a significant difference in recurrence between SPGD and SDD. Additionally, five more studies were included in the present meta-analysis [19–23], and no significant effect on the recurrence rate was reported in these newly included studies. Compared to a recently published meta-analysis [39], we updated four studies (3 cohort studies [19, 21, 22] and 1 RCT [20]) with a total of 735 participants. Therefore, we performed an updated analysis with the four newly published studies. Besides, compared to the recent meta-analysis, further subgroup analysis based on different complications between the two drainage methods was conducted in the present study. The pooled data showed that there were no significant differences in mortality, which was consistent with previous studies [35–39]. Meanwhile, the present study proved that there was no significant difference in the incidence of postoperative infection, epilepsy and pneumocephalus between the two drainage methods. Additionally, in the present meta-analysis, a subgroup analysis of the SPGD group was performed, revealing no significant differences in recurrence rates between the SPD and SGD groups, although there was substantial heterogeneity among the studies (I^2^ = 55%). This may be because active aspiration was performed in both groups in the study by Sjåvik et al. [24] but not in other studies. Further analysis based on the three RCTs included in the present study (Soleman et al., Pathoumthong et al., and Kariaperumal et al.) [9, 20, 25] showed that there was no significant difference in recurrence rate, mortality and incidence of surgical complications between the SDD group and the SPGD group.

Our study had some limitations. First, the studies included in the present analysis were mostly retrospective or prospective. Therefore, any conclusions drawn are limited by their respective research designs, including memory and observation biases. In addition, the duration of follow-up varied among studies. Subacute and late complications may be more evident in studies with longer follow-up times, highlighting the need for additional long-term analyses. Besides, many of the outcomes in the present analysis were only reported by a few of the studies included. Future research may focus more on this issue.

## Conclusion

The present results demonstrate that the choice of SDD vs. SPGD has no significant effect on the prognosis of patients with CSDH. Thus, SPGD may be an alternative treatment option for CSDH. Further studies with larger sample sizes are warranted to confirm this finding.

## Supporting information

S1 Appendix PRISMA-P checklist. (DOC)

S2 Appendix Search strategy. (DOCX)

S3 Appendix Newcastle-Ottawa quality assessment scale. (DOCX)
