# Supplementary material for: Subperiosteal/subgaleal drainage vs. subdural drainage for chronic subdural hematoma: A meta-analysis of postoperative outcomes
Source: PLoS One. 2023 Aug 1;18(8):e0288872. doi: 10.1371/journal.pone.0288872 (PMC10393133; doi:10.1371/journal.pone.0288872)
Supplement: S2 Appendix — (DOCX) [file pone.0288872.s002.docx]

**Pubmed**

| **Search** | **Query** |
| --- | --- |
| #1 | "Drainage"[Mesh] |
| #2 | Drain [Title/Abstract] |
| #3 | #1 OR #2 |
| #4 | "Hematoma, Subdural, Chronic"[Mesh] |
| #5 | (Subdural Hematoma, Chronic [Title/Abstract]) OR (Chronic Subdural Hematoma[Title/Abstract]) OR (Chronic Subdural Hematomas[Title/Abstract]) OR (Hematoma, Chronic Subdural[Title/Abstract]) OR (Hematomas, Chronic Subdural[Title/Abstract]) OR (Subdural Hematomas, Chronic[Title/Abstract]) OR (Hemorrhage, Subdural, Chronic[Title/Abstract]) |
| #6 | #4 OR #5 |
| #7 | #3 AND #6 |
| Items:550 | |

**Embase**

| **Search** | **Query** |
| --- | --- |
| #1 | 'drainage'/exp |
| #2 | 'drain':ab,ti |
| #3 | #1 OR #2 |
| #4 | 'subdural hematoma'/exp |
| #5 | 'hematoma, subdural, chronic':ab,ti |
| #6 | 'subdural hematoma, chronic':ab,ti |
| #7 | 'chronic subdural hematoma':ab,ti |
| #8 | 'chronic subdural hematomas':ab,ti |
| #9 | 'hematoma, chronic subdural':ab,ti |
| #10 | 'hematomas, chronic subdural':ab,ti |
| #11 | 'subdural hematomas, chronic':ab,ti |
| #12 | 'hemorrhage, subdural, chronic':ab,ti |
| #13 | #4 OR #5 OR #6 OR #7 OR #8 OR #9 OR #10 OR #11 OR #12 |
| #14 | #3 AND #13 |
| Items: 159 | |

**Cochrane Library**

| **Search** | **Query** |
| --- | --- |
| #1 | MeSH descriptor: [Drainage] explode all trees |
| #2 | (Drain):ti,ab,kw (Word variations have been searched) |
| #3 | #1 OR #2 |
| #4 | (Hematoma, Subdural, Chronic):ti,ab,kw (Word variations have been searched) |
| #5 | (Subdural Hematoma, Chronic):ti,ab,kw (Word variations have been searched) |
| #6 | (Chronic Subdural Hematoma):ti,ab,kw (Word variations have been searched) |
| #7 | (Chronic Subdural Hematomas):ti,ab,kw (Word variations have been searched) |
| #8 | (Hematoma, Chronic Subdural):ti,ab,kw (Word variations have been searched) |
| #9 | (Hematomas, Chronic Subdural):ti,ab,kw (Word variations have been searched) |
| #10 | (Subdural Hematomas, Chronic):ti,ab,kw (Word variations have been searched) |
| #11 | (Hemorrhage, Subdural, Chronic):ti,ab,kw (Word variations have been searched) |
| #12 | #4 OR #5 OR #6 OR #7 OR #8 OR #9 OR #10 OR #11 |
| #13 | #3 AND #12 |
| Items:66 | |
