# Supplementary material for: Subperiosteal/subgaleal drainage vs. subdural drainage for chronic subdural hematoma: A meta-analysis of postoperative outcomes
Source: PLoS One. 2023 Aug 1;18(8):e0288872. doi: 10.1371/journal.pone.0288872 (PMC10393133; doi:10.1371/journal.pone.0288872)
Supplement: S3 Appendix — (DOCX) [file pone.0288872.s003.docx]

| Study | Selection | | | | Comparability | Outcome | | | |
| --- | --- | --- | --- | --- | --- | --- | --- | --- | --- |
|  | Representative-ness of the intervention cohort | Selection of the non-  intervention cohort | Ascertainment of intervention | Demonstration that outcome of interest was not present at start of study | Comparability of cohorts on the basis of the design or analysis | Assessment of outcome | Was follow up long enough for outcomes to occur | Adequacy of follow up of cohorts | Aggregate score |
| Hwang | 1 | 1 | 1 | 1 | 1 | 0 | 1 | 1 | 7 |
| Singh | 1 | 0 | 1 | 1 | 1 | 0 | 1 | 1 | 6 |
| Kamenova | 1 | 0 | 1 | 0 | 1 | 0 | 0 | 1 | 4 |
| Gazzeri | 1 | 1 | 1 | 1 | 1 | 0 | 1 | 1 | 7 |
| Zhang | 1 | 1 | 1 | 1 | 1 | 0 | 1 | 1 | 7 |
| Häni | 1 | 1 | 1 | 0 | 1 | 0 | 1 | 1 | 6 |
| Glancz | 0 | 1 | 1 | 0 | 1 | 0 | 1 | 1 | 5 |
| Sjåvik | 1 | 1 | 1 | 1 | 1 | 1 | 1 | 1 | 8 |
| Ishfaq | 1 | 1 | 1 | 0 | 1 | 0 | 1 | 1 | 6 |
| Chih | 0 | 1 | 1 | 0 | 1 | 0 | 1 | 1 | 5 |
| Oral | 1 | 1 | 1 | 0 | 1 | 0 | 1 | 1 | 6 |
| Bellut | 1 | 1 | 1 | 0 | 1 | 1 | 1 | 1 | 7 |
